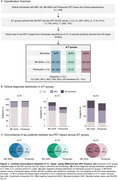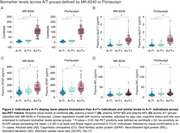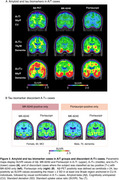# PART characterization using MK‐6240 and Flortaucipir

**DOI:** 10.1002/alz70856_106723

**Published:** 2026-01-11

**Authors:** Carolina Soares, Emma Ruppert, Pamela C.L. Ferreira, Marina Scop Madeiros, Andreia Rocha, Matheus Scarpatto Rodrigues, Bruna Bellaver, Markley Silva Oliveira, Guilherme Povala, Livia Amaral, Firoza Z Lussier, Joseph C. Masdeu, Dana L Tudorascu, Thomas K Karikari, David N. Soleimani‐Meigooni, Juan Fortea, Val J Lowe, Hwamee Oh, Belen Pascual, Brian A. Gordon, Pedro Rosa‐Neto, Suzanne L. Baker, Tharick A Pascoal

**Affiliations:** ^1^ University of Pittsburgh, Pittsburgh, PA, USA; ^2^ Pontifical Catholic University of Rio Grande do Sul, Porto Alegre, RS, Brazil; ^3^ Houston Methodist Research Institute, Houston, TX, USA; ^4^ University of Gothenburg, Mölndal, Sweden; ^5^ University of California, San Francisco, San Francisco, CA, USA; ^6^ Sant Pau Memory Unit, Hospital de la Santa Creu i Sant Pau, Biomedical Research Institute Sant Pau, Barcelona, Spain; ^7^ Mayo Clinic, Rochester, MN, USA; ^8^ Brown University, Providence, RI, USA; ^9^ Washington University in St. Louis, School of Medicine, St. Louis, MO, USA; ^10^ McGill University, Montreal, QC, Canada; ^11^ Lawrence Berkeley National Laboratory, Berkeley, CA, USA; ^12^ University of Pittsburgh School of Medicine, Pittsburgh, PA, USA

## Abstract

**Background:**

Older adults with brain tau pathology but no evident amyloid‐beta (A) pathology can be referred to as primary age‐related tauopathy (PART). However, in vivo characterization of PART using biomarkers remains unclear, varying based on the method used to define tau pathology (e.g. different tau‐PET tracers). Here, we conducted a head‐to‐head characterization of PART using two different tau‐PET tracers.

**Method:**

We studied 433 individuals from the HEAD cohort (244 CU, 138 MCI, and 51 with dementia). Participants underwent clinical assessments, MRI, A‐PET, both MK‐6240 and Flortaucipir scans, and a subset with plasma biomarkers (*n* = 332). A‐PET positivity was defined as Centiloid>24. Tau‐positivity(T) was determined by MK‐6240 and Flortaucipir SUVR values exceeding the mean+2SD in at least one Braak region anchored in CU A‐ individuals followed by visual confirmation in A‐/T+ cases. Concordance across biomarkers was assessed among PART (A‐/T+), A‐/T‐ and A+/T+ groups. Plasma GFAP, *p*‐tau217, NFL levels, and Centiloids were compared across A/T groups using linear regression.

**Result:**

We found a total of 25 (5.8 %) cases classified as A‐/T+ defined by at least one tau tracer (74±6.3 years, 52% females, 17 CU, 3 MCI, 5 with dementia). Detection of A‐/T+ cases varied by tracer: MK‐6240(*n* = 20), Flortaucipir(*n* = 16) (Figure 1A). Among A‐/T+ individuals, MK‐6240 identified 13 CU, 4 MCI, and 3 dementia cases, while Flortaucipir identified 11 CU, 3 MCI, and 2 dementia cases(Figure 1B). Concordance between MK‐6240 and Flortaucipir in A‐/T+ cases was low (*n* = 11, 44%)(Figure 1C). A‐/T+ individuals showed no significant biomarker differences from A‐/T‐ but had lower biomarker levels than A+/T+: Centiloids (MK‐6240: T=15.08, *p* <0.001; Flortaucipir: T=14.36, *p* <0.001), GFAP (MK‐6240: T=5.04, *p* < 0.01; Flortaucipir: T=2.92, *p* <0.001), *p*‐tau217 (MK‐6240: T=7.77, *p* <0.001; Flortaucipir: T=6.34, *p* <0.001), except for NFL (MK‐6240: T=1.35, *p* = 0.18; Flortaucipir: T=1.14, *p* = 0.25)(Figure 2). Representative A and tau biomarker cases (Figure 3A), along with discordant cases classified as T+ by only one tracer (Figure 3B), are illustrated.

**Conclusion:**

Our preliminary analysis revealed a higher prevalence of A‐/T+ cases with MK‐6240 than Flortaucipir, low concordance between tracers and consistently lower biomarker levels in A‐/T+ compared to A+/T+. Larger studies and longitudinal analyses are necessary to ascertain the pathological trajectories of A‐/T+ individuals with varying tau biomarker profiles.